# Exosomes Derived from *M. Bovis* BCG Infected Macrophages Activate Antigen-Specific CD4^+^ and CD8^+^ T Cells *In Vitro* and *In Vivo*

**DOI:** 10.1371/journal.pone.0002461

**Published:** 2008-06-18

**Authors:** Pramod K. Giri, Jeffrey S. Schorey

**Affiliations:** Department of Biological Sciences, Eck Center for Global Health and Infectious Diseases, University of Notre Dame, Notre Dame, Indiana, United States of America; Johns Hopkins School of Medicine, United States of America

## Abstract

Activation of both CD4^+^ and CD8^+^ T cells is required for an effective immune response to an *M. tuberculosis* infection. However, infected macrophages are poor antigen presenting cells and may be spatially separated from recruited T cells, thus limiting antigen presentation within a granuloma. Our previous studies showed that infected macrophages release from cells small membrane-bound vesicles called exosomes which contain mycobacterial lipid components and showed that these exosomes could stimulate a pro-inflammatory response in naïve macrophages. In the present study we demonstrate that exosomes stimulate both CD4^+^ and CD8^+^ splenic T cells isolated from mycobacteria-sensitized mice. Although the exosomes contain MHC I and II as well as costimulatory molecules, maximum stimulation of T cells required prior incubation of exosomes with antigen presenting cells. Exosomes isolated from *M. bovis* and *M. tuberculosis* infected macrophages also stimulated activation and maturation of mouse bone marrow-derived dendritic cells. Interestingly, intranasal administration of mice with exosomes isolated from *M. bovis* BCG infected macrophages induce the generation of memory CD4^+^ and CD8^+^ T cells. The isolated T cells also produced IFN-γ upon restimulation with BCG antigens. The release of exosomes from infected macrophages may overcome some of the defects in antigen presentation associated with mycobacterial infections and we suggest that exosomes may be a promising *M. tuberculosis* vaccine candidate.

## Introduction

Granulomas are required for controlling an *M. tuberculosis* (Mtb) infection and the site of a chronic inflammatory response. Granuloma formation requires CD4^+^ T cells as mice deficient in these cells fail to form granulomas or control the infection [1]. Initial activation of CD4^+^ T cells occurs within the draining lymph node and involves class II MHC antigen presentation by *M. tuberculosis*-infected dendritic cells (DCs) as well as exposure to DC-derived IL-12p70 [2] [3]. Activated CD4+ T cells migrate to the site of infection and produce cytokines and chemokines, especially IFN-γ, which serves to recruit and activate macrophages promoting granuloma formation and the inflammatory process [4]. Maintaining the granuloma and controlling the infection requires continued antigen presentation mediated by antigen presenting cells (APCs) within the granuloma. CD8^+^ T cells are also required for maintaining an effective immune response at later stages of infection [5] [4]. This requires processing of mycobacterial antigens and presentation onto class I MHC by cells within the granuloma.

Mtb has multiple mechanisms to inhibit antigen processing and presentation within an infected macrophage [6]. Quantitative analysis by Gercken *et al.* indicates that monocytes cocultured with Mtb for 6 days showed a 3- to 10-fold decrease in their ability to stimulate Th1 cell proliferation when compared with uninfected controls [7]. However, granulomas from Mtb infected humans and mice maintain significant CD4^+^ T cell activation and IFN-γ production [8] [9]. Moreover, there is significant activation of CD8^+^ T cells within the granuloma [10] despite the sequestration of the Mtb within a phagosome and away from the class I antigen processing pathway. Analysis of the cellular architecture within a human granuloma also indicates a spatial separation between the Mtb and the CD8^+^ T cells [11].

Despite the data indicating that infected macrophages are relatively poor presenters of Mtb antigens to both CD8^+^ and CD4^+^ T cells, there is a robust acquired immune response associated with an Mtb infection which implies that alternative mechanisms of antigen presentation may be functioning within a granuloma. In this report we identify a previously unknown mechanism for antigen cross-priming in the context of a mycobacterial infection. Previous studies have shown that DCs, macrophages and other cell types release small 30 to 100 nm vesicles. This exocytic process involves fusion of multivesicular bodies (MVBs) with the plasma membrane and subsequent release of the intraluminal vesicles as exosomes [12]. The membranes of the MVBs have their origin within the endocytic network and thus exosomes consist of proteins and lipids present within this network. Exosomes released from mycobacterial-infected macrophages contain bacterial components and stimulate a MyD88-dependent proinflammatory response in naïve macrophages [13]. The isolated exosomes also contain both class I and II MHC as well as costimulatory molecules. Interestingly, prior studies have indicated that exosomes containing tumor antigens are potent stimulators of tumor-specific T cells *in vitro* and *in vivo* [14]. Therefore in this study we tested the hypothesis that exosomes released from *M. bovis* BCG infected macrophages contain mycobacterial antigens which could stimulate a T cell response. We found that exosomes released from BCG infected macrophages upon intranasal administration of C57Bl/6 mice induced a CD4^+^ and CD8^+^ memory T cell response. We also found that exosomes released from BCG infected macrophages could stimulate DC activation and maturation *in vitro*. Together the data demonstrates a potential mechanism for antigen cross-priming during a mycobacterial infection and suggest that isolated exosomes, which have both antigenic and adjuvant characteristics, may be an alternative tuberculosis vaccine candidate.

## Results

### Presence of the Ag85 complex on exosomes from *M. bovis* BCG-infected J774 cells

Studies by David Russell and colleagues demonstrated that macrophages infected with *M. bovis* BCG release vesicles containing mycobacterial lipids [15] [16]. In previous studies we also observed that vesicles released from macrophages infected with *M. avium*, BCG or *M. tuberculosis* contain LAM, glycopeptidolipids, and the19 kDa lipoprotein. Flow cytometric analysis of these vesicles indicated that they contained exosome-associated proteins including CD86, CD80, LAMP1, LAMP2 and MHC class I and II [13]. Importantly, the vesicles were negative for FCR-γII/III and annexin-V staining which are known markers of apoptotic vesicles [13].

Both mycobacterial lipid and protein components have been shown to be antigenic [17]. We previously reported that exosomes released from infected macrophages contain mycobacterial glycolipids; however, protein composition was not evaluated in these studies [13]. Therefore we purified exosomes released from BCG-infected macrophages as described previously [13] and probed the exosomes using antibodies against HBHA, α-crystallin, PstS1 and antigen 85 complex (Ag85). Although exosomes from BCG infected macrophages did not have detectable levels for HBHA, α-crystallin or PstS1, we did observe a strong band of the correct size for Ag85 in our Western blot (data not shown). Using a polyclonal antibody made against Mtb whole cell lysate (minus LAM), we observed ∼10 protein bands on Western blot specific to exosomes isolated from BCG infected macrophages (data not shown).

### Activation of T cells by exosomes from BCG-infected macrophages

**Figure 1:**
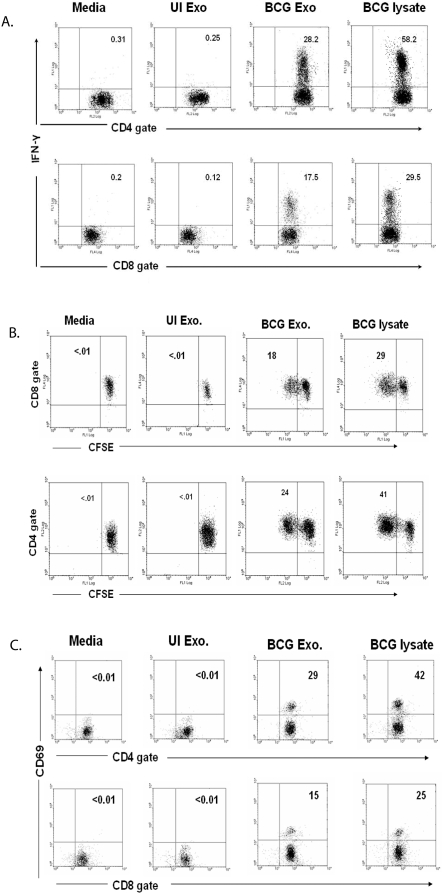
Activation of BCG sensitized splenic T cells by exosomes from BCG-infected macrophages. Balb/C mice were infected with BCG (1×10^6^/mouse). Four weeks post-infection, splenocytes were harvested. (A) Splenocytes from BCG infected mice were cultured with BCG or UI exosomes, BCG lysate or media. 12 hr post-treatment, cells were stained with anti-CD4-PE, anti-CD8-PE-Cy5 and anti-IFN-γ-FITC and analyzed by flow cytometry. (B) Splenocytes from BCG infected mice, labeled with 2.5 µM CFSE, were treated with BCG or UI exosome, BCG lysate or media. 72 hr post-treatment, cells were stained with anti-CD4-PE or anti-CD8-PE-Cy5 and analyzed by flow cytometry. (C) Splenocytes from BCG infected mice were cultured with BCG or UI exosomes, BCG lysate or media. 72 hr post-treatment, cells were stained with anti-CD4-PE, anti-CD8-FITC and anti-CD69-PE-Cy5 and analyzed by flow cytometry. Total lymphocytes were gated using FSC/SSC. CFSE, CD69 and intracellular IFN-γ levels were detected on gated CD4^+^ and CD8^+^ lymphocytes. Results are shown as percent positive. Cells were negative for staining with isotype control antibodies. Results are representative of at least 3 independent experiments.

Exosomes from infected J774 cells contain mycobacterial antigens as well as class I and II MHC and CD86 suggesting that these vesicles may be capable of inducing an antigen-specific T cell response. This possibility is further strengthened by previous studies showing that exosomes containing tumor antigens can stimulate antigen-specific T cell responses [18] [19]. Therefore we incubated exosomes from BCG infected or uninfected J774 cells with total splenocytes isolated from BCG infected mice. As shown in figure 1A, exosomes from infected cells induced intracellular IFN-γ expression in both CD8^+^ and CD4^+^ T cells while exosomes from uninfected cells failed to induce IFN-γ. We found 28% and 17.5% of the total CD4^+^ and CD8^+^ T cells respectively, produced IFN-γ upon exosome treatment suggesting that exosomes have a significant antigenic component for both T cell populations. T cell activation by exosome treatments was further evaluated by measuring cell proliferation and CD69 expression. Cells were labeled with the fluorescent dye CFSE prior to exosome treatment as described in Material and Methods. As the labeled cells proliferate, the CFSE staining diminishes. Splenic cells obtained from BCG-infected mice were treated with exosomes and as shown in figure 1B, CD4^+^ and CD8^+^ T cells showed diminished CFSE staining upon treatment with exosomes from infected but not from uninfected J774 cells. CD69 expression, which is a marker of T cell activation [20], was also elevated in T cells treated with exosomes from BCG infected J774 cells (Fig. 1C).

### Exosome-mediated activation of T cells requires presentation by APCs

**Figure 2:**
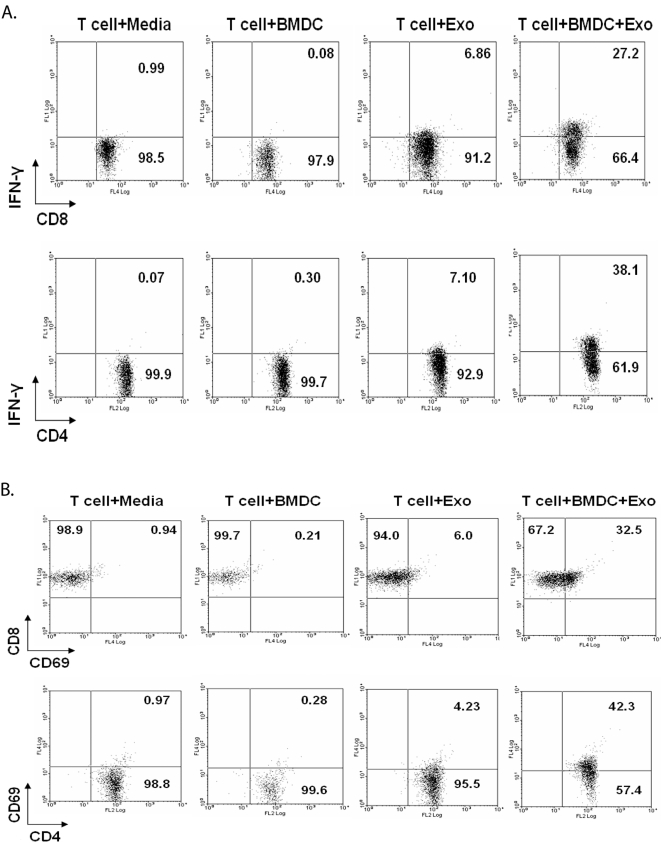
APCs are required for optimal T cell stimulation by exosomes. BMDCs were generated from Balb/C bone marrow cells and CD3^+^ T cells were purified from the spleens of BCG infected mice. Purified CD3^+^ T cells (5×10^5^) were cultured alone or in presence of BMDCs (1×10^5^) with or without BCG exosomes. Cells were cultured for 12 hr and stained with anti-CD4-PE, anti-CD8-PE-Cy5 and anti-IFN-γ-FITC (A) or cultured for 72 hr and stained with anti-CD4-PE, anti-CD8-FITC and anti-CD69-PE-Cy5 (B). Cells were analyzed by flow cytometry. Total lymphocytes were gated using FSC/SSC and intracellular IFN-γ and CD69 levels were detected on gated CD4^+^ and CD8^+^ lymphocytes. Results are shown as percent positive. Cells were negative for staining with isotype control antibodies. Results are representative of at least 2 independent experiments.

Exosomes bearing tumor antigens have been shown to induce a tumor-specific T cell response. However, it is less clear whether exosomes can induce T cell activation directly or require uptake and subsequent presentation of exosome-derived antigens by APCs; although the most recent data supports the latter [21]. To address this question in the context of exosomes from BCG infected cells, CD3+ T cells were isolated from the spleens of BCG infected mice and treated with exosomes in the presence or absence of mouse bone marrow-derived DCs. As shown in figure 2, intracellular IFN-γ as well as CD69 expression were increased modestly in both CD4^+^ and CD8^+^ T cells when the cells were treated with exosomes alone compared to cells treated with media alone. However, levels of IFN-γ and CD69 expression by T cells were considerably elevated when DCs were present during the incubation, suggesting that uptake and presentation by APCs is required for optimal stimulation.

### Activation of T cells *in vivo* by exosomes from BCG-infected macrophages

**Figure 3:**
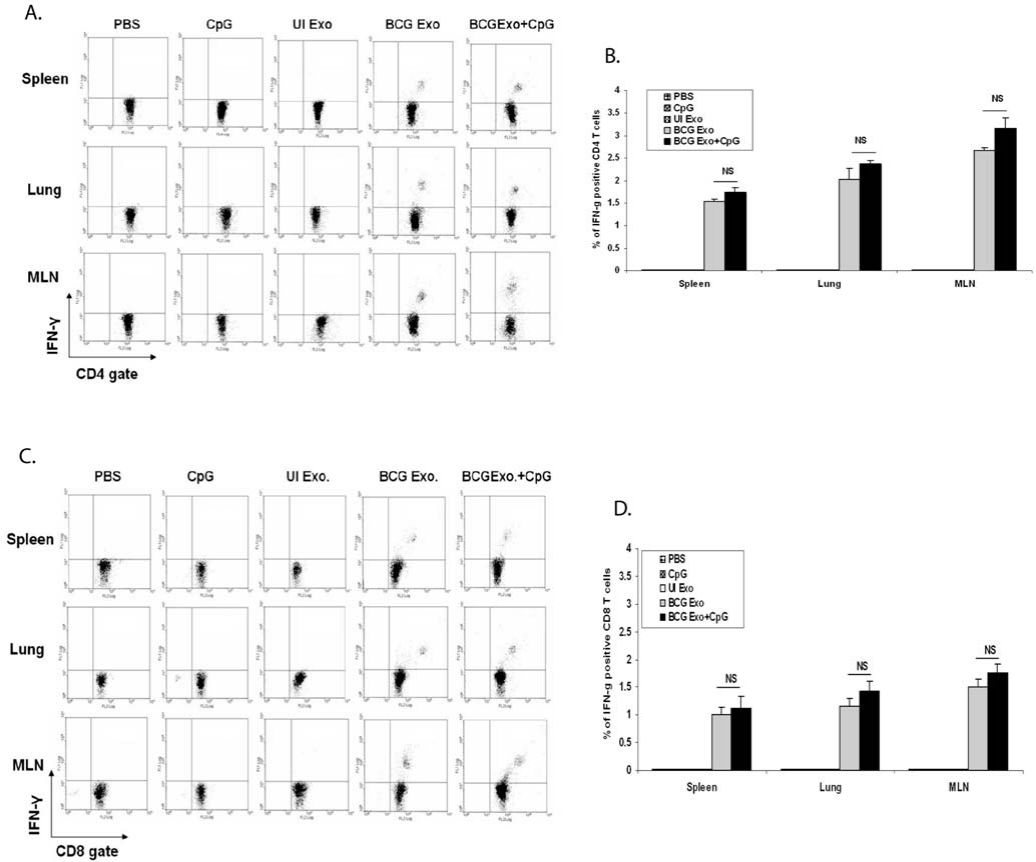
Exosomes from BCG infected macrophages induce antigen-specific IFN-γ producing T cells. Lymphocytes from lungs, MLN and spleens were isolated from mice intranasally injected with exosomes or PBS and cultured in the presence or absence of BCG lysate. Cells were stained with anti-CD4-PE, anti-CD8-PE and anti-IFN-γ-FITC and analyzed by flow cytometry. (A) T cells stained for CD4 and intracellular IFN-γ. (B) percentage of total CD4^+^ T cells which expressed IFN-γ. Values are mean±SD of two independent experiments. (C) T cells stained for CD8 and intracellular IFN-γ (D) Percentage total CD8^+^ T cells which expressed IFN-γ. Values are mean±SD of two different experiments. Total lymphocytes were gated using FSC/SSC and intracellular IFN-γ levels were detected on gated CD4^+^ and CD8^+^ lymphocytes. Cells were negative for staining with isotype control antibodies. Exo- exosomes, UI- uninfected. NS-not significant.

To determine if exosomes could activate naïve T cells *in vivo*, C57Bl/6 mice were treated intranasally with exosomes +/− the adjuvant CpG. Two weeks after the final dose, mice were sacrificed and their spleens, lungs and mediastinal lymph nodes (MLN) were isolated. Single cell suspensions from the various organs were stimulated *ex vivo* with BCG lysate. As shown in Figures 3A and 3B, there was a small but significant population of CD4^+^ T cells from all three organs which produced IFN-γ upon restimulation with BCG antigens. As expected, this was specific to mice treated with exosomes from BCG-infected macrophages as exosomes from uninfected cells failed to stimulate IFN-γ production. The activation of T cells was not limited to CD4^+^ cells as exosomes were also able to stimulate a CD8^+^ T cell response as defined by IFN-γ production upon restimulation with BCG antigens (Fig. 3C and D). Interestingly, the addition of the adjuvant CpG only marginally increased the effectiveness of the exosomes. This contrasts with what is observed for subunit vaccines using mycobacterial proteins, as the activation of a T cell response requires the addition of an adjuvant [22]. As a control we also isolated T cells from intranasally BCG infected mice and stimulated the T cells *in vitro* with BCG antigens. As anticipated, there was a more robust IFN-γ response as 5.8%, 14% and 12% of the CD4^+^ T cells in the spleen, lung and MLN respectively were positive for cytokine production.

**Figure 4:**
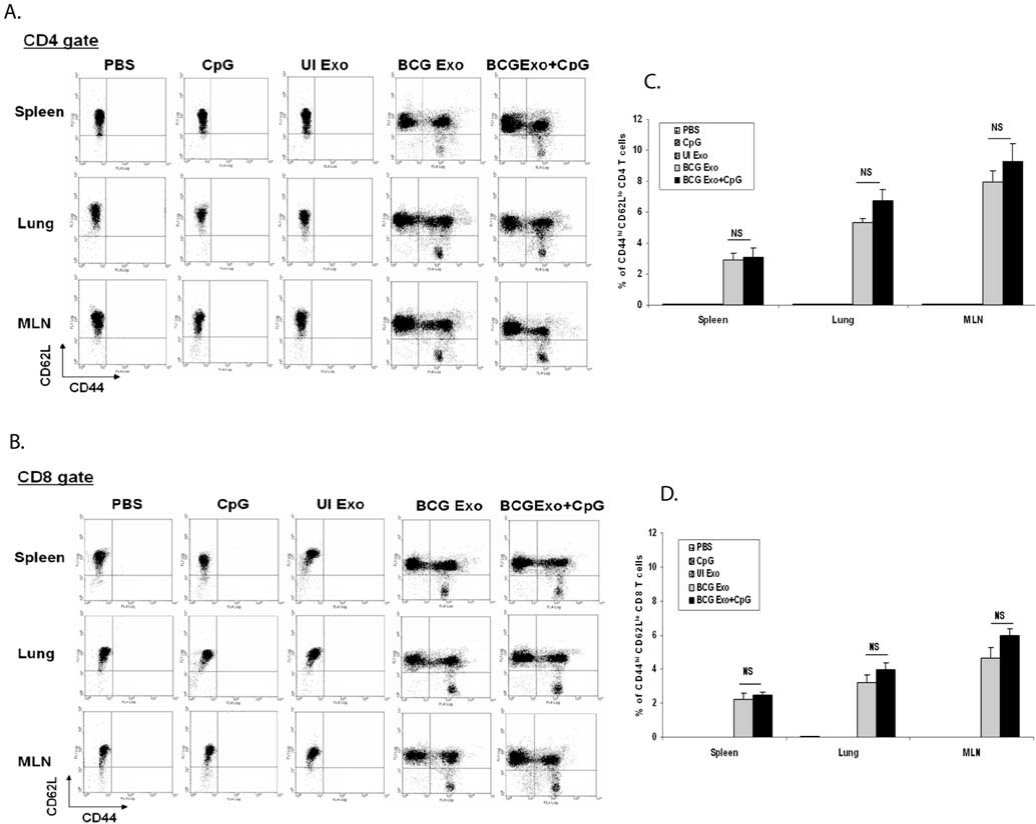
Exosomes from BCG infected macrophages induced effector memory T cells *in vivo*. Lymphocytes from lungs, MLN and spleens were isolated from mice intranasally injected with exosomes or PBS and cultured in the presence or absence of BCG lysate. Cells were stained with anti-CD4-PE, anti-CD8-PE, antiCD44-PECy5, and antiCD62L-FITC and analyzed by flow cytometry. (A) Expression of effector memory CD4^+^ T cells (CD44^hi^ and CD62L^low^). (B) Percentage of total CD4^+^ T cells which were CD44^hi^ and CD62L^low^. Values are mean±SD of two different experiments. (C) Expression of memory CD8^+^ T cells (CD44^hi^ and CD62L^low^). (B) Percentage of total CD8^+^ T cells which were CD44^hi^ and CD62L^low^. Values are mean±SD of two different experiments. Total lymphocytes were gated using FSC/SSC and intracellular IFN-γ levels were detected on gated CD4^+^ and CD8^+^ lymphocytes. Cells were negative for staining with isotype control antibodies. Exo- exosomes, UI- uninfected. NS-not significant.

An effective tuberculosis vaccine requires the development of memory CD4^+^ and CD8^+^ T cells. Therefore, to determine if exosomes could induce a memory response, mice were intranasally treated with the different exosome preparations +/− CpG adjuvant and organs were harvested as described above. As shown in figures 4A and B, exosomes from BCG-infected but not from uninfected macrophages induced a population of effector memory T cells (CD62L-low, CD44-high) in the spleen, lung and MLN. A similar result was obtained for CD8^+^ T cells (Fig. 4C and D). As observed for IFN-γ production, the presence of CpG plus exosomes resulted in only a marginal increase in the number of CD62L^low^, CD44^high^ effector memory T cells compared to exosomes alone. In contrast, mice vaccinated intranasally with BCG produced significantly higher numbers of effector memory CD4^+^ T cells with percent positive being 11%, 7.1% and 21.2% in the spleen, lung and MLN respectively.

### Exosomes from Mtb- and BCG-infected macrophages stimulate DC maturation and activation

**Figure 5:**
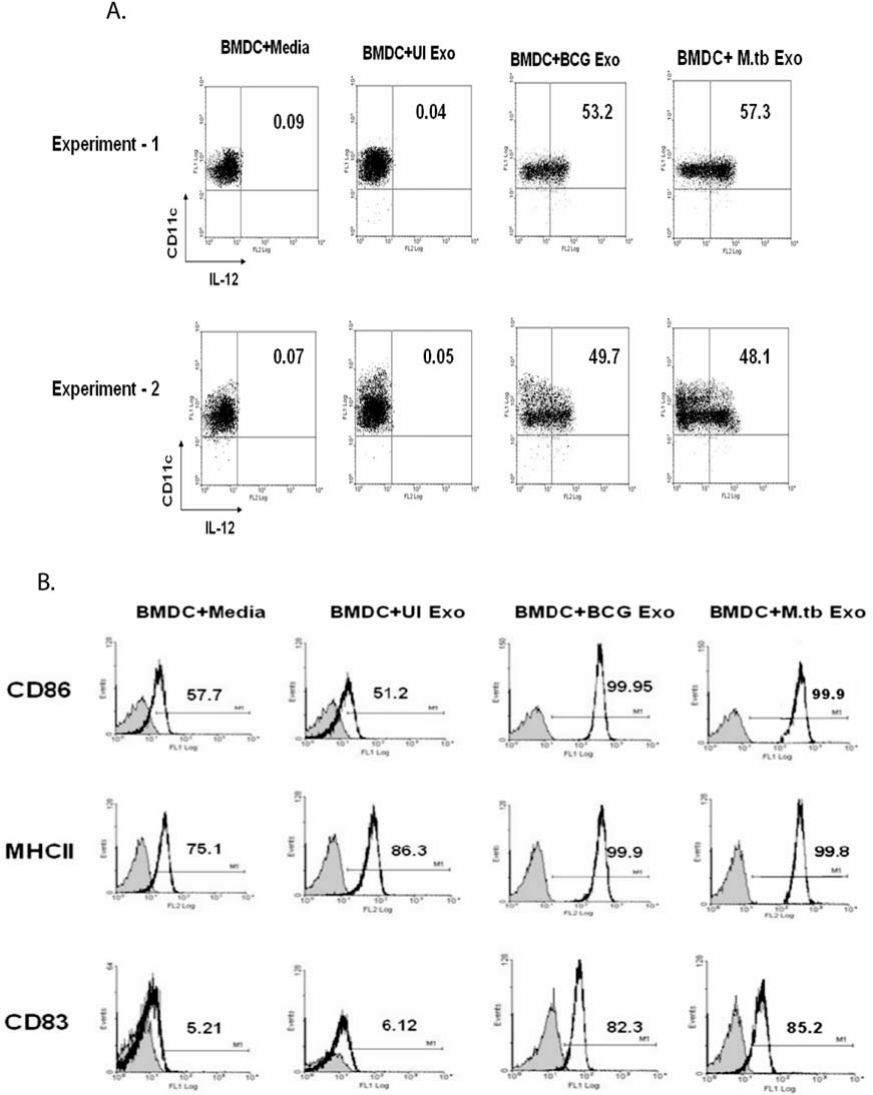
Activation and maturation of BMDC by exosomes from Mtb- and BCG-infected macrophages. (A) BMDCs following a 12 hr treatment with exosomes from Mtb, BCG or uninfected (UI) macrophages were labeled with anti-CD11c-FITC and anti-IL-12p40-PE and analyzed by flow cytometry. (B) BMDCs were cultured in the presence or absence of exosome and 24 hr post-treatment, CD86, MHC II and CD83 expression was evaluated by flow cytometry. Results are shown as percent positive. The shaded area indicates staining with isotype control antibodies. Results are representative of at least 2 independent experiments.

Since exosomes were shown to stimulate a T cell response in the absence of adjuvant, we tested their ability to activate mouse bone marrow-derived DC. Intracellular IL-12p40 was measured as this cytokine is required for stimulating a T_H_1 response and is a classic marker of DC activation. As expected, non-activated DCs were positive for CD11c and negative for intracellular IL-12p40 (Fig. 5A). The same profile was seen for DCs treated with exosomes isolated from uninfected J774 cells. In contrast, ∼50 to 60% of the DCs treated with exosomes from Mtb or BCG-infected cells were positive for intracellular IL-12p40 staining (Fig. 5A). To address DC maturation, the cells were analyzed for CD83, CD86 and MHC class II expression. As shown in figure 5B, treatment of DCs with exosomes isolated from infected but not from uninfected cells resulted in a marked increase in expression for all three markers. We did not observe any clear differences between exosomes isolated from Mtb- or BCG-infected macrophages in their ability to activate DCs or induce DC maturation. This suggests that potential differences between exosomes derived from Mtb- and BCG-infected macrophages do not affect their ability to stimulate DC maturation and activation.

## Discussion

*M. tuberculosis* as well as other mycobacterial species shed or release both lipid and protein components during growth in broth culture and in macrophages [23] [24]. Some of the shed mycobacterial components including the Ag85 complex and ESAT6 are known to be immunogenic and have been used effectively as subunit vaccines [25]. However, the accessibility of these proteins to the immune system during an *in vivo* infection is less clear. A number of studies have indicated that infected DCs and macrophages are poor presenters of mycobacterial antigens [26]. Nevertheless, a robust T cell response to shed mycobacterial components is observed during an *in vivo* Mtb infection in mice [27] as well as in tuberculosis patients [9]. This suggests that alternative mechanisms for presenting mycobacterial antigens may be functioning *in vivo*. One previously described mechanism for cross-presentation of Mtb antigens in the context of CD8^+^ T cells is through release of apoptotic blebs. Work by Schaible *et al.* demonstrated that apoptotic vesicles obtained from Mtb infected macrophages contain mycobacterial antigens and can stimulate MHC class I and CD1b restricted T cells. Engulfment of the apoptotic vesicles by DC was required for cross-presentation to T-cells isolated from a mycobacteria-sensitized donor [28]. Nevertheless, macrophages infected with pathogenic strains of Mtb show minimal apoptosis, thus limiting this potential mechanism for activating antigen-specific CD8^+^ T cells [29] [30]. Surprisingly, recent data suggest that Mtb can escape from the phagosome into the cytoplasm within infected macrophages and DCs and therefore the released mycobacterial antigens may be exposed to the class I antigen presentation pathway [31]. Here we describe an alternative mechanism for presentation of mycobacterial antigens through exosomes released from infected cells and we hypothesize that this mechanism of cross-presentation could function *in vivo* to facilitate an acquired immune response. We have shown that exosomes activate CD8^+^ T cells, indicating that antigens present on exosomes can be processed through the class I MHC presentation pathway. We predict that these exosomes may be responsible, at least in part, for the robust CD8^+^ T cell response seen during the course of an Mtb infection.

Prior studies have shown that exosomes can function to modulate immune responses, including immune stimulation and immune suppression [12]. Tumor-derived exosomes, which are enriched in tumor antigens, are a novel source of antigens for promoting CTL cross-priming. Immature DCs secrete exosomes and can transfer functional MHC-peptide complexes to activated DCs. Consistent with a stimulatory role in the immune response, exosomes secreted by DCs which carry tumor antigens can elicit T cell activation *in vivo* [32] [33]. Recent studies have also shown that exosomes containing microbial antigens can protect against infection. Studies by Colino and Snapper showed that injecting mice with exosomes containing *Streptococcus pneumoniae's* capsular polysaccharide type 14 cross-reactive antigen induced a protective antibody response [34]. Mice treated with exosomes derived from DCs pulsed with *Toxoplasma* antigens was also shown to protect the mice against subsequent infection [35].

Consistent with these studies, we found that exosomes from *M. bovis* BCG infected macrophages can stimulate proliferation and IFN-γ production by pre-sensitized CD4^+^ and CD8^+^ T cell. The antigens responsible for this restimulation remain to be defined but the Ag85 complex is likely one candidate, as at least one member of this complex is present on exosomes. Members of the Ag85 complex are known to be highly immunogenic [23]. Moreover, mycobacterial lipids present on exosomes may also function to stimulate a T cell response through CD1. The restimulation of T cells by exosomes was significantly enhanced in the presence of APCs. This suggests that the exosomes function more in cross-priming then in direct activation of T cells.

Whether an effective vaccine can be generated using exosomes containing mycobacterial antigens awaits further investigation. However, previous studies have shown a strong correlation between expression of IFN-γ producing CD4^+^ and CD8^+^ T cells and vaccine effectiveness [36]. Moreover, the development of effector memory cells is also associated with protection [37] [38]. Our results show that intranasal administration of exosomes from BCG-infected macrophages could activate naïve T cells and that these T cells respond with IFN-γ production upon restimulation with BCG antigens *ex vivo*. These exosomes also induced the development of effective memory CD4^+^ and CD8^+^ T cells. Therefore, our data suggest that exosomes derived from BCG-infected macrophage could function as an effective vaccine. Nevertheless, additional experiments are needed to further dissect the phenotype of the T cells activated upon exosome treatment and most importantly, determine if intranasal administration of exosomes isolated from Mtb- or BCG-infected DCs or macrophages protects against an Mtb infection.

In summary, the present study indicates that exosomes isolated from *M. bovis* BCG infected macrophages can stimulate sensitized CD4^+^ and CD8^+^ T cells *in vitro* as well as induce naïve T cell activation *in vivo*. Based on the present study and previous data indicating that exosomes from mycobacteria-infected macrophages contain TLR ligands and are pro-inflammatory, we hypothesize that exosomes could promote both innate and acquired immune responses within the granuloma but distal to the infection foci. Thus cross-priming by exosomes may help promote the robust immune response seen in infected individuals. Moreover, we suggest that exosomes may be a viable candidate for a tuberculosis vaccine as they have both adjuvant and antigenic qualities.

## Materials and Methods

### Bacteria culture, complement opsonization and mycobacterial infections

*M. bovis* BCG and *M. tuberculosis* H_37_R_V_ (Mtb) stocks were generated as described [13]. Appropriate concentrations of mycobacteria were suspended in Dulbecco's modified Eagle's medium (DMEM, Gibco) containing 10% normal horse serum as a source of complement components, followed by a 2 hr incubation at 37°C. The mouse macrophage cell line J774 was maintained at 37°C in 5% CO_2_ in DMEM supplemented with 10% fetal bovine serum (FBS) (Life Technologies), 25 mM Na^+^-HEPES, 100-U/ml penicillin and 100-µg/ml streptomycin (BioWhittaker, Walkersville, MD). Infection of macrophages with complement-opsonized BCG and Mtb was carried out 3–4 hr after seeding the cells and infected for 4 hrs as described [39]. An MOI of 20 was used to reach approximately 80% of the cells infected.

### Mouse infections

Balb/c mice 6–8 weeks of age were used for this study (Harlan, Indianapolis IN). The mice were housed at the Frieman Life Science Center, University of Notre Dame and all mouse experiments were approved by the Institutional Animal Care and Use Committee. For BCG infection, *M. bovis* BCG was grown as described above. At the time of infection, BCG bacilli were thawed and centrifuged at 1200g for 15 min. The pellet was washed thrice with sterile PBS, sonicated for 30 sec. and passed through 27G needle 10 times. Finally, bacilli were suspended in sterile PBS at the concentration of 2×10^7^ bacilli/ml. *M. bovis* BCG was administered via the retro-orbital intravenous route at a single dose of 10^6^ CFU/mouse in 50 µl of PBS. For some group, BCG was administered via intranasal route. For intranasal infection, mice were lightly anesthetized with isoflurane and 30 µl volume containing 1×10^6^ bacilli in PBS were administered dropwise to external nares of the mice (15 µl per nostril) using a micropipette. As a control group, mice were injected with sterile PBS only. The infection experiments complied with the Institutional Animal Care and Use Committee guidelines.

### Isolation and Purification of Exosomes

Exosomes were isolated and purified as described previously [13]. Briefly, for exosome production, cell culture media containing FCS was centrifuged at 100,000g for 15 hrs to remove any contaminating exosomes. Culture supernatants were collected from BCG or uninfected J774 cells after 72 hr and cell debris were removed by centrifugation at 300*g* for 10 min at 4°C. Cleared culture supernatants were filtered through 0.22-µm polyethersulfone filters (Corning, NY, USA). Exosomes are 30–90 nm in diameter and filter freely through 0.22-µm filters. Filtered supernatants were centrifuged at 10,000*g* for 30 min, and the exosomes pelleted from the supernatant at 100,000*g* for 1 h at 4°C. Exosomes were purified using a sucrose gradient where, the 100,000g pellet was re-suspended in 0.5 ml of 0.25 M sucrose (20 mM HEPES/NaOH, pH 7.2) and layered on top of a linear sucrose gradient (0.25–2.0 M sucrose, 20 mM Hepes/NaOH, pH 7.2). The resulting sample was centrifuged at 100,000g for 15 hr. Gradient fractions (7×1.5 ml) were collected from the top of the tube, diluted with 10 ml of PBS and ultracentrifuged at 100,000g for 1 hr. The purified exosomes from BCG-infected, Mtb-infected or uninfected cells were resuspended in PBS to a protein concentration of approximately 0.1 to 0.5 mg/ml as determined by a Micro BCA assay (Pierce, Rockford, IL). Exosomes were sterilized by filtration through 0.22 µM syringe sterile filter (Millipore) and stored at −80°C.

### Electron microscopy

Exosome pellets were resuspended and fixed in PBS containing 2% glutaraldehyde and then loaded onto Formar/carbon-coated electron microscopy grids. The samples were contrasted with uranyl acetate to visualize membrane, and viewed with a Hitachi H-600 TEM microscope (Hitachi, USA).

### Detection of mycobacterial components associated with exosomes

Mycobacterial components on exosomes were detected by Western blot. Briefly, 8 µg of exosomes were loaded onto 14% SDS-PAGE gels, electrophoresed, and transferred onto polyvinylidene difluoride membranes (PVDF) (Milipore, Bedford, MA). The membranes were probed with appropriate dilution of monoclonal antibodies against Ag85 complex (CS-90), PstS1 (IT-23), α-crystallin (IT-20), HBHA (α-HBHA), LAM (CS-35), 19 kDa lipoprotein (IT-12) and polyclonal antibodies against whole cell lysate minus LAM (E-293). This was followed by incubation with HRP-conjugated secondary antibodies, and detected using an enhanced chemiluminescence kit (Roche Diagnostic, USA). All antibodies against mycobacterial components were obtained from Colorado State University, Colorado, USA, under TB Vaccine Testing and Research Materials Contract (NIH-NIAID NO1-A1-40091).

### Generation of bone marrow-derived dendritic cells (BMDCs) and treatment with exosomes

Bone marrow cells were isolated from the tibias and femurs of Balb/C mice as described previously [39]. Contaminating RBCs were lysed with ACK lysis buffer (150 mM NH_4_Cl, 1 mM KHCO_3_ and 0.1 EDTA pH 7.3). Cells were cultured in complete RPMI -1640 containing GM-CSF (200 U/ml). After 12 days in culture, flow analysis revealed that >85% cells were positive for CD11c. To evaluate the maturation and activation of BMDCs by exosome treatment, BMDCs were seeded in 24 well plates at 1×10^5^ cells/well in 0.5 ml of exosome-free culture media for 3 hr and then treated with exosomes from BCG, Mtb or uninfected macrophages (50 µg/ml). After 24 hr cells were washed with FACS buffer and the expression of MHC II, CD86 and CD83 were determined by flow cytometry using fluorochrome tagged specific antibodies according to manufacturer instructions. For intracellular IL-12p40 staining, cells were treated with BCG exosomes and UI exosomes for 12 hr and adding 2 µM monensin (Biolegend) in the last 6 hr of infection. Treated cells were first stained with anti-CD11c-FITC, permeabilized with permeabilization buffer (Biolegend), then stained with anti-IL-12p40-PE (BD Pharmingen, CA), washed with FACS buffer, and analyzed by flow cytometry.

### Treatment of mice with exosomes

Exosomes were administered to mice in three doses at two week intervals via an intranasal route. At each time points, C57Bl/6 mice were lightly anesthetized with isofluorane and 30 µl volume containing 25 µg of exosomes from BCG infected and uninfected macrophages, +/− 30 µg CpG-ODN (Invitrogen) adjuvant in PBS were administered dropwise to external nares of the mice (15 µl per nostril) using a micropipette. As a positive control, BCG was administered as a single dose of 1×10^6^ CFU/mouse in 30 µl of PBS via intranasal route as described above. Negative control groups received only CpG-ODN adjuvant and PBS.

### Isolation of lymphocytes from spleen and mediastinal lymph node

Spleens from BCG infected mice were isolated after 30 days of infection. For isolation of spleen and mediastinal lymph nodes (MLN) from exosome-treated or untreated animals, mice were sacrificed two weeks after the final immunization and spleens and MLNs were aseptically removed. The spleens were then perfused with RPMI-1640 using 10 ml syringe fitted with 26G needle to obtain a single cell suspension of splenocytes. MLN cells were perfused by the pistol of a 3 ml sterile dispovan syringe. The splenocytes and MLN cell suspension were then centrifuged at 300×g for 10 min. The RBCs were lysed by hypotonic shock using 3 ml ACK lysis buffer for 5 min. The cells were then washed thrice with RPMI 1640 to remove lysed RBCs and lysis buffer.

### Isolation of Lung lymphocytes

Lung lymphocytes from exosome treated and untreated as well as infected mice were isolated as described previously [40] with minor modifications. Pooled lungs were homogenized in 6 ml sterile complete RPMI-1640 in a glass homogenizer and incubated at 37°C for 2 h with type IV collagenase (125 to 150 U/ml) and DNase I (50–60 U/ml). Digested lung tissue was then pressed through a 70-µm nylon mesh and centrifuged at 300g for 10 min. The cell pellet was then resuspended in complete RPMI-1640 media. The cell suspension was layered on a histoplaque. Density centrifugation was carried out for 10 min at 300×*g* at room temperature. Lung mononuclear cells were obtained from the interface, washed, counted, and resuspended at 10^6^ cells/ml in complete RPMI-1640 media.

### *In vitro* T cell assays

For Carboxy Fluoroscein Succinimidyl Ester (CFSE) labeling, splenocytes from infected and control mice were labeled with CFSE (2.5 µM), and 1×10^6^ cells/well were seeded in 24-well tissue culture plates in 500 µl of RPMI-1640 (supplemented with 10% heat inactivated fetal calf serum, 25 mM HEPES, 2 mM L-glutamine, 1 mM sodium pyruvate, 100 IU/ml penicillin and 100 µg/ml streptomycin) in the presence or absence of BCG exosomes or UI exosomes (50 µg/ml). Phytohemagglutinin (PHA) (5 µg/ml) was used as a positive control for cell viability. The cells were incubated for 72 hr at 37°C, 5% CO_2_. Dilution of CFSE after 72 hr was analyzed by FACS. For CD69 expression, splenocytes were cultured in the presence or absence of BCG exosomes or uninfected exosomes (50 µg/ml) for 72 hr as indicated above. Cells were labeled with anti-CD69-PE-Cy5 (Biolegend), anti-CD4-PE (Biolegend) and anti-CD8-PE-Cy5 (Biolegend) according to manufacturer instructions, washed and analyzed by flow cytometry. For intracellular IFN-γ, cells were treated with exosomes for a total of 12 hrs with the final 6 hours in presence of 2 µM monensin. These cells were first stained with anti-CD4-PE and anti-CD8-PE-Cy5 then permeabilized with permeabilization buffer (Biolegend), and stained with anti-IFN-γ-FITC (Biolegend BD pharmingen, San Diago CA) according to manufacturer instructions, washed with FACS buffer and analyzed by flow cytometry. For intracellular IFN-γ staining of lymphocytes from lung, spleen, and MLN of exosomes treated and untreated mice were incubated with BCG lysate (10 µg/ml) for a total of 12 hrs with the final 6 hours in presence of 2 µM monensin. These cells were first stained with anti-CD4-PE and anti-CD8-PE then permeabilized with permeabilization buffer (Biolegend), and stained with anti-IFN-γ-FITC (Biolegend) according to manufacturer instructions, washed with FACS buffer and analyzed by flow cytometry.

T cells were purified from splenocytes of BCG infected animals by T cell specific negative selection on magnetic cell sorting (MACS) columns (Miltenyi Biotech) using Pan T cell isolation kit (Miltenyi Biotech) according to manufacturer instructions. T cells (5×10^5^) were cultured together with autologous BMDCs (1×10^5^) in the presence or absence of BCG exosomes or uninfected exosomes (50 µg/ml). Intracellular IFN-γ and CD69 expression were analyzed 12 and 72 hr post-exosome treatment respectively as described above.

### Analysis of memory phenotype (CD44^hi^ and CD62L^low^) on CD4^+^ and CD8^+^ T cells

Tri-color flow cytometry was done for analysis of memory phenotype. Two weeks after the final immunization or 30 days after BCG infection, lungs, MLNs and spleen cells were isolated from exosomes treated and untreated animals. Cells were washed with FACS wash buffer and incubated on ice with panel of antibodies: antiCD4-PE, antiCD8-PE, antiCD44-PECy5, antiCD62L-FITC at the concentration of 0.5 µg/million cells in 50 µl of FACS buffer for 30 min at 4°C. Cells were then washed and fixed with 2% paraformaldehyde and analyzed by flow cytometry. All the analysis was done with an acquisition of 100,000 events. Gating of CD4^+^ and CD8^+^ cells in the lymphocyte-rich regions was based on forward-angle light scatter and log Phycoerythrin fluorescence (FL2-H) and then the expression of CD62L (FL1-H) and CD44 (FL4-H) on CD4^+^ or CD8^+^ gated lymphocytes were analyzed. Isotype controls for cells stained with antibodies were analyzed at each time point.

### Statistical Analysis

Wherever applicable, data were analyzed using the Microsoft Excel Software program. Group comparisons were performed by using the unpaired Student's *t* test.
